# Metabolic Profiling Analysis of Patients With Coronary Heart Disease Undergoing Xuefu Zhuyu Decoction Treatment

**DOI:** 10.3389/fphar.2019.00985

**Published:** 2019-09-10

**Authors:** Tianqi Tao, Tao He, Xiaoreng Wang, Xiuhua Liu

**Affiliations:** Department of Pathophysiology, Chinese PLA General Hospital, Beijing, China

**Keywords:** metabolomics, coronary heart disease, Xuefu Zhuyu decoction, LC-MS/MS, ethnopharmacology

## Abstract

Coronary heart disease (CHD) remains the leading cause of morbidity and mortality worldwide. Traditional Chinese medicine (TCM) is one of the effective complementary and alternative therapies used to improve the prognosis of CHD patients. Xuefu Zhuyu (XFZY) decoction, a classical traditional Chinese medication for regulating Qi and promoting blood circulation, has a clinical benefit in CHD; however, the underlying mechanism is not clear. Recently, it was found that the metabolites involved in amino acid metabolism and the tricarboxylic acid cycle were altered in CHD patients with Qi and Yin deficiency syndrome. To understand the material foundation of Qi, it is of great significance to study the differential metabolites involved in Qi during treatment of CHD with Qi-regulating and blood-promoting herbs. In this study, we investigated the metabolic profiles of serum in CHD patients by nontargeted metabolomics analysis to detect differential metabolites between the XFZY decoction group and placebo group. Ten CHD patients were enrolled and treated with placebo granules or XFZY decoction granules in a random and double-blind manner. Serum samples of all patients were evaluated by untargeted high-performance liquid chromatography with tandem mass spectrometry-based metabolomics. In total, 513 metabolites were detected in the serum of CHD patients, and six of these metabolites participating in seven metabolic pathways were significantly different between CHD patients treated with XFZY decoction and the placebo group. Among the six differential metabolites, FA (20:2)-H and tetracarboxylic acid (24:0), involved in fatty acid metabolism; cis-aconitic acid, which participates in the tricarboxylic acid cycle; 2-deoxy-D-glucose, involved in glucose metabolism; and N-acetylglycine, involved in amino acid metabolism, were decreased, whereas spermine, which participates in amino acid metabolism, was increased as compared with the placebo group. Our findings, combined with the perspective of biological functions, indicate that 2-deoxy-D-glucose and spermine might constitute the partial material foundation of Qi in CHD patients treated with XFZY decoction.

## Introduction

Coronary heart disease (CHD) remains the major cause of morbidity and mortality worldwide, responsible for about one in every seven deaths (Lozano et al., 2012). It is predicted to continue until 2030, accounting for 14% of all deaths (Mirzaei et al., 2009). Currently, the incidence of CHD is still on the rise and is associated with a high mortality rate, despite the use of effective Western medicine treatments. Therefore, effective complementary and alternative therapy is necessary to improve functional status and quality of life in CHD patients. Traditional Chinese medicine (TCM) integrated with Western medicine in the treatment of CHD has made great progress. As an effective complementary and alternative therapy, TCM has improved the prognosis of CHD patients. According to the TCM theory, Qi is the commander of blood. Qi stagnation causes blood stasis, which leads to heart vessel blockage stasis. CHD with Qi stagnation and blood stasis is a common syndrome of CHD. Therefore, one of the critical treatments of CHD in TCM is to regulate Qi and promote blood circulation (Xu and Chen, 2007). Xuefu Zhuyu (XFZY) decoction, which originated from the ancient Chinese document “Yilin Gaicuo” in the late Qing Dynasty, has effects on regulating Qi and promoting blood circulation, which is the basic prescription for the treatment of CHD (Meng et al., 2018). Previous studies confirmed that XFZY decoction reduced the incidence of cardiovascular events and improved the prognosis of patients with CHD; however, the underlying mechanism is still unclear.

CHD is characterized by an atherosclerotic plaque-induced narrowing of the coronary arteries, which results in myocardial ischemia, infarction, and postinfarction heart failure. The abnormal substrate and energy metabolism induced by myocardial ischemia is fundamental in the development of CHD (Doenst et al., 2013). Because complete metabolism of glucose is more oxygen efficient than that of fatty acids (FA; Mjos, 1971), myocardial ischemia causes the limitation of FA oxidation and the effective utilization of glucose catabolism, which may lead to the decrease of adenosine triphosphate (ATP) production. In addition, several additional pathways that do not lead to ATP generation, such as the pentose phosphate pathway and the hexosamine biosynthetic pathway, are activated. Therefore, metabolic abnormality plays a key role in affecting the development and prognosis of CHD (Nicholson et al., 1999; Kordalewska and Markuszewski, 2015). Recently, it was found that metabolites involved in amino acid metabolism and tricarboxylic acid (TCA) cycle might partially constitute the material foundation of Qi, according to the Qi and blood theory of TCM (Zhou et al., 2019). Therefore, we studied differential metabolites in the serum of CHD patients to explore the effects of TCM on regulating Qi and promoting blood circulation. It is of great significance to elucidate the mechanism of improving the prognosis of CHD patients through Qi-regulating and blood-promoting herbs and to study Qi and blood in TCM theory.

Metabolomics is one of the youngest branches of “-omics” (such as genomics, transcriptomics, proteomics) techniques to offer the most up-to-date insight into the state of the system in the field of systems biology. It contains approaches to detect low-molecular-weight metabolites (molecular mass <1,500 Da), which reflects changes in the final representations of an organism’s phenotype. In our study, we used nontargeted metabolomics analysis to focus on the differential metabolites in the serum from CHD patients treated with placebo and XFZY decoction. We aimed to provide references for the pathogenesis of CHD and the treatment of Qi-regulating and blood-promoting drugs, which refined the understanding of Qi and blood in TCM theory.

## Materials and Methods

### Participants

In this study, a total of 10 CHD patients were recruited from the Chinese PLA General Hospital from April to July 2017. On the basis of routine Western medicine treatment according to the guidelines, participants were treated with placebo granules and XFZY decoction granules randomly and double blindly (twice a day for 12 weeks). The study was approved by the Ethics Review Committee of Chinese PLA General Hospital (No. S2015-048-01) and registered at www.chictr.org.cn (registration number ChiCTR-IOR-15006989).

Inclusion criteria included age <75 years and compliance with the diagnostic criteria for stable CHD according to the American College of Cardiology (ACC)/American Heart Association (AHA) guidelines in 2014 (Fihn et al., 2014). All participation was voluntary, and patients signed an informed agreement. We excluded patients with severe renal dysfunction (serum creatinine >220 μmol/l in males or >175 μmol/l in females), liver dysfunction (aspartate aminotransferase or alanine aminotransferase level three times higher than normal), uncontrolled blood pressure, diabetes mellitus, hemorrhagic diseases, malignant tumors, autoimmune diseases, or hematological or psychiatric diseases; pregnant women; and those who were allergic to components of the research drugs. We also excluded patients with a history of myocardial infarction, severe chronic heart failure, severe arrhythmia, or a cardiac pacemaker. Patients were treated with routine Western medicine according to the guidelines, including anti-ischemia drugs (β receptor blocker or calcium antagonist), antiplatelet drugs (aspirin or clopidogrel), anticoagulant drugs (heparin or low-weight-molecular heparin), and lipid-lowering drugs (statins). Complications, such as hypertension and dyslipidemia, were treated according to relevant guidelines. During the study, nitroglycerin was used to relieve acute angina pectoris. The specifications of nitroglycerin are 0.5 mg/tablet. The original treatment was maintained during the trial.

### TCM Treatment

Both XFZY decoction granules and placebo granules were prepared and standardized from China Resources Sanjiu Medical & Pharmaceutical Company (Shenzhen, China). XFZY decoction granules refer to the proportion of components of XFZY decoction, and the botanical compositions are shown in Table 1. The voucher specimens of total compositions were stored by China Resources Sanjiu Medical & Pharmaceutical Company (Shenzhen, China). The chemical compositions of XFZY decoction mainly consisted of ferulic acid, paeoniflorin, amygdalin, hydroxysafflor yellow A, catalpol, platycodin D, liquiritin, and ammonium glycyrrhizinate, as indicated by the high-performance liquid chromatography (HPLC) profile of the extract performed by the manufacturer (**Supplementary Figure 1**). The chemical structures of those major compounds are depicted in **Supplementary Figure 2**. The main components of placebo granules were caramel pigments (4 g), and maltodextrin (1,000 g). Using the technical requirements of the quality standards of TCM formula granules, a mixing solution was dried, and granules were then formed. Quality control (QC) of the pharmaceutical process was carried out in accordance with Pharmaceutical Production Quality Management Standards (2015).

**Table 1:** Botanical compositions of Xuefu Zhuyu decoction.

Herb (local name)	Medicinal parts	Amount in application (g)
*Prunus persica* (L.) Batsch (Tao Ren)	Seed	12
*Angelica sinensis* (Oliv.) Diels (Dang Gui)	Root	15
*Conioselinum anthriscoides* “Chuanxiong” (Chuan Xiong)	Root	10
*Carthamus tinctorius* L. (Hong Hua)	Flower	10
*Paeonia lactiflora* Pall. (Chi Shao)	Root	10
*Rehmannia glutinosa* (Gaertn.) DC. (Di Huang)	Root	15
*Citrus × aurantium* L. (Zhi Qiao)	Fruit	6
*Bupleurum chinense* DC. (Chai Hu)	Root	3
*Platycodon grandiflorus* (Jacq.) A.DC. (Jie Geng)	Root	4.5
*Achyranthes bidentata* Blume (Niu Xi)	Root	9
*Glycyrrhiza uralensis* Fisch. ex DC. (Gan Cao)	Root	6

All components in Xuefu Zhuyu decoction granules were fully validated using http://mpns.kew.org/mpns-portal/?_ga=1.111763972.1427522246.1459077346.

### Randomization, Control, and Double Blinding

Subjects were randomly grouped by researchers using a random-number table by SAS statistical software. The allocation ratio between the XFZY decoction group and placebo group was 1:1. Drugs were coded and packaged according to random numbers. The blind bottom could not be disassembled during the trial. A placebo group was established as the control of XFZY decoction to exclude the effect of the placebo on CHD treatment. The participants, staff, and researchers were blinded to the treatment group allocation.

### Sample Collection and Preparation

All subjects fasted overnight, and 4 ml of peripheral venous blood was drawn in the morning of the day of the consultation. The blood was then coagulated for 30 min at 4°C and centrifuged at 3,000 × *g* for 15 min. The serum supernatant was collected. We then added 400 μl of prechilled methanol to 100 μl serum samples to precipitate the proteins. The mixture was shaken for 15 s, incubated at –80°C for 1 h, and then centrifuged at 13,400 × *g* for 20 min at 4°C. The supernatant was transferred to a new Eppendorf tube and dried before storage in the –80°C freezer. A pooled QC sample solution was prepared by combining equal volumes of serum from each sample and treated using the same procedure. This sample was used to monitor the reliability of the entire experiment. One QC sample was inserted in front of the batch of experimental samples, and each QC sample was inserted in an interval of 10 to 15 samples. Once a QC sample ended, the instrumental stability was monitored throughout the batch process (Li et al., 2018).

### Untargeted LC-MS/MS Analysis and Metabolite Identification

Samples were analyzed by untargeted liquid chromatography with tandem mass spectrometry (LC-MS/MS) using an Ultimate 3000 UHPLC (Dionex) system combined with a Thermo Q-Exactive (Orbitrap) mass spectrometer (Thermo Fisher Scientific, San Jose, CA, USA). Data identifications were performed by Trace Finder (Thermo Fisher Scientific, San Jose, CA, USA). First, metabolites were potentially identified by accurate masses according to the endogenous MS database. Meanwhile, metabolites that matched with the spectra in the fragment database were confirmed at the MS/MS level. The mass tolerances of primary and secondary identifications were 10 and 15 ppm, respectively. Moreover, a 0.25-min retention time shift was applied (Ning et al., 2018).

### Data Processing and Statistical Analysis

SIMCA 14.0 software (Umetrics AB, Umea, Sweden) was used for multivariate statistical analysis. Unsupervised principal component analysis (PCA) was employed to assess the quality, homogeneity, outlier identification, and dominating trends of the group separation inherent in the data set. A supervised orthogonal partial least squares discriminant analysis (OPLS-DA) was applied to distinguish between the classes and to identify the differential metabolites. Variable importance in the projection (VIP) value was generated in PLS-DA model. The quality of the multivariate statistical analysis model was evaluated by R^2^X and Q^2^. Student’s *t* test was used to determine the difference in metabolites between the two groups. Metabolites with VIP > 1 and *P* < 0.05 were considered to be the most probable metabolites, which could be used to analyze the difference between the two groups and to assess the severity of CHD. Subsequently, the internal metabolite MS/MS database was used to identify metabolites by matching accurate quality and MS/MS spectra. MetaboAnalyst 4.0 (http://www.metaboanalyst.ca/; Wishart Research Group, McGill University, Canada) was used to show differential metabolites with a heat map and to predict the metabolic pathways (Chong et al., 2018). SPSS v13.0 (Chicago, IL, USA) was used for statistical analysis. Student’s *t* test was performed for two-group comparisons on baseline characteristics. One-way analysis of variance was used to detect the homogeneity of variance. Values are presented as mean ± SD. *P* < 0.05 was considered statistically significant.

## Results

### Baseline Characteristics

Ten CHD patients were randomly and double blindly divided into two groups: five patients were treated with placebo granules for 12 weeks, and five patients were treated with XFZY decoction granules for 12 weeks. Serum samples from 10 CHD patients were detected by nontargeted metabolomics analysis. The baseline characteristics of the subjects are shown in Table 2 and include age, body mass index (BMI), blood pressure (systolic pressure, diastolic pressure), heart rate, five kinds of blood routine indices [including red blood cell (RBC), hemoglobin (HGB), white blood cell (WBC), neutrophil (NE), and platelet (PLT)], 10 kinds of biochemical indices [including alanine aminotransferase (ALT), aspartate aminotransferase (AST), serum creatinine (SCr), blood urea nitrogen (BUN), uric acid (UA), triglyceride (TG), total cholesterol (TC), low-density lipoprotein (LDL), high-density lipoprotein (HDL), and fasting glucose (Glu)], and four kinds of coagulation parameters [including prothrombin time (PT), activated partial thrombin time (APTT), fibrinogen (FIB), and thrombin time (TT)]. The average ages of the CHD patients in the two groups were 54.4 ± 5.73 years and 57.0 ± 15.84 years, respectively. The mean BMIs of the two groups were 28.26 ± 6.62 kg/m^2^ and 28.49 ± 3.71 kg/m^2^, respectively. With the statistical analysis by Student’s *t* test, the levels of HGB and Glu were increased, whereas APTT was decreased in the XFZY decoction group as compared with those in the placebo group (*P* < 0.05). However, all values were within the normal range. There was no significant difference in other characteristics between the two groups (*P* > 0.05).

**Table 2:** Baseline characteristics of patients.

	Placebo (n = 5)	XFZY (n = 5)	*P* Value
Gender (F/M)	2/3	1/4	0.690
Age, years	54.4 ± 5.73	57 ± 15.84	0.739
BMI, kg/m^2^	28.26 ± 6.62	28.49 ± 3.71	0.946
Blood pressure, mmHg			
SBP	126.80 ± 15.40	133.60 ± 11.61	0.453
DBP	86.80 ± 6.42	82 ± 8.37	0.339
Heart rate, beats/min	67.20 ± 5.22	71.40 ± 15.19	0.575
Library data			
RBC, ×10^12^/L	4.78 ± 0.08	5.12 ± 0.42	0.153
HGB, g/L	140.4 ± 7.57	159 ± 16.02	0.047*
WBC, ×10^9^/L	6.03 ± 1.98	7.08 ± 2.34	0.465
NE, %	64.94 ± 9.80	63.99 ± 4.54	0.849
PLT, ×10^9^/L	231 ± 47.43	231.6 ± 92.69	0.990
AST, U/L	20.18 ± 4.98	20.86 ± 7.06	0.865
ALT, U/L	22.80 ± 10.51	27.40 ± 8.86	0.476
SCr, μmoI/L	68.34 ± 23.38	82.08 ± 15.89	0.309
UA, μmoI/L	319.74 ± 90.24	319.84 ± 24.85	0.998
BUN, mmoI/L	4.63 ± 1.20	5.31 ± 1.23	0.399
TG, mmol/L	2.22 ± 1.15	1.32 ± 0.38	0.135
CHOL, mmol/L	3.07 ± 1.59	4.48 ± 0.68	0.106
HDL, mmol/L	1.29 ± 0.48	1.18 ± 0.12	0.630
LDL, mmol/L	1.94 ± 0.47	2.70 ± 0.74	0.088
Glu, mmol/L	4.83 ± 0.37	5.64 ± 0.59	0.031*
PT, s	13.18 ± 0.63	12.52 ± 0.98	0.242
APTT, s	37.84 ± 1.63	32.94 ± 3.14	0.015*
FIB, g/L	2.84 ± 0.35	3.19 ± 0.44	0.201
TT, s	16.68 ± 0.66	16.72 ± 1.58	0.960

Values are mean ± SD. *P < 0.05 versus placebo group.

### Analysis of QC Samples

We randomly selected 10 serum samples from CHD patients for metabolomics testing (five in the placebo group and five in the XFZY decoction group). In our research, we used a customized database and nontargeted metabolomics methods to analyze the data. From data collection to data analysis of clear compounds, we quantitatively identified hundreds of compounds according to the established Orbitrap workflow. A total of 513 metabolites were detected in the samples after treatment, which were identified with known MS/MS information. Samples were then subjected to PCA and OPLS-DA analysis.

### Serum Metabolomics and Pathway Analysis

**Figure 1:**
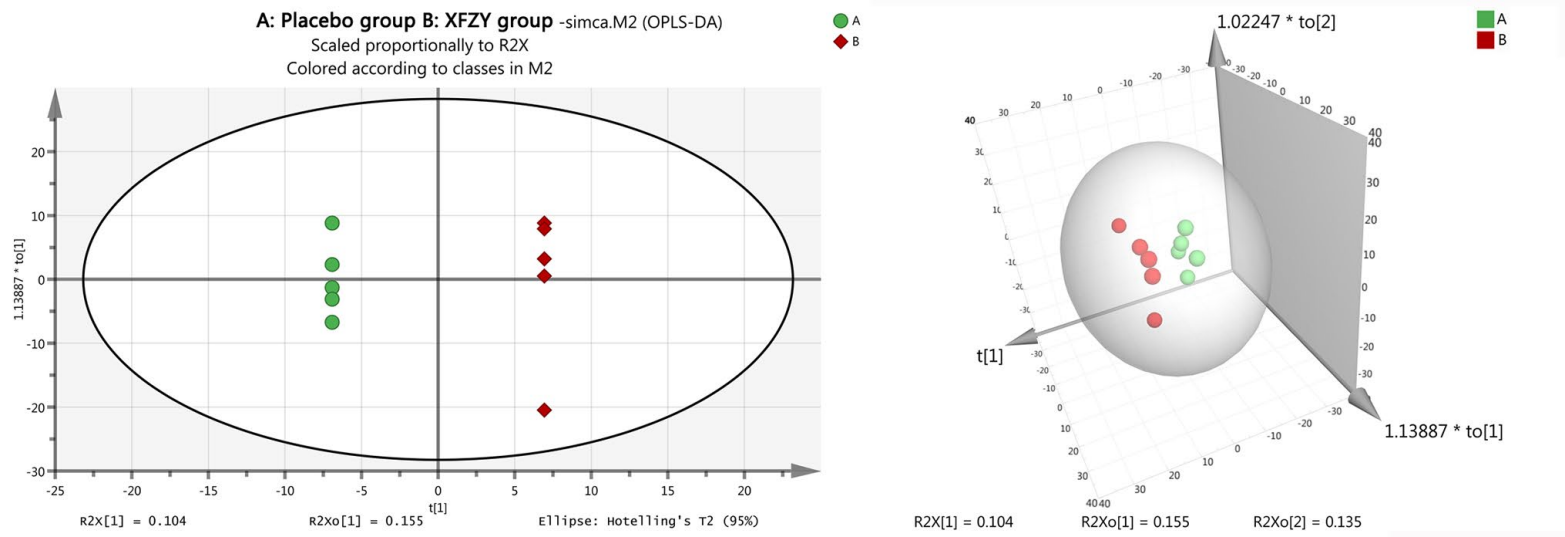
The orthogonal projection to latent structure-discriminant analysis (OPLS-DA) score plots compared the Xuefu Zhuyu decoction group to the placebo group. The left image is the two-dimensional graph of the OPLS-DA score plots, and the right image is the three-dimensional graph of the OPLS-DA score plots. The placebo group is shown in green, and the Xuefu Zhuyu decoction group is shown in red.

To evaluate the identification ability of 513 metabolites, we analyzed the metabolic differences between the two groups, merged all identified compounds (513 in total), and imported them into SIMCA-P software for PCA. The results showed that there was no significant difference between the two groups (*P* > 0.05). Further analysis with two- or three-dimensional orthogonal partial least -squares discriminant method obtained an OPLS-DA score chart (**Figure 1**), which confirmed that there was significant difference between the XFZY decoction group and the placebo group. The OPLS-DA model established a good model and made an accurate prediction (R^2^X = 76.2% and Q^2^ = 39.1%).

After filtering 513 different metabolites in each group with the requirements of VIP > 1 and *P* < 0.05, six differential metabolites in the XFZY decoction group were changed as compared with the placebo group, namely, tetracosanoic acid (24:0), N-acetylglycine, FA (20:2)-H, 2-deoxy-D-glucose (2-DG), cis-aconitic acid, and spermine. Compared with the placebo group, tetracosanoic acid (24:0), N-acetylglycine, FA (20:2)-H, 2-DG, and cis-aconitic acid were downregulated, whereas spermine was upregulated in the XFZY decoction group.

**Figure 2:**
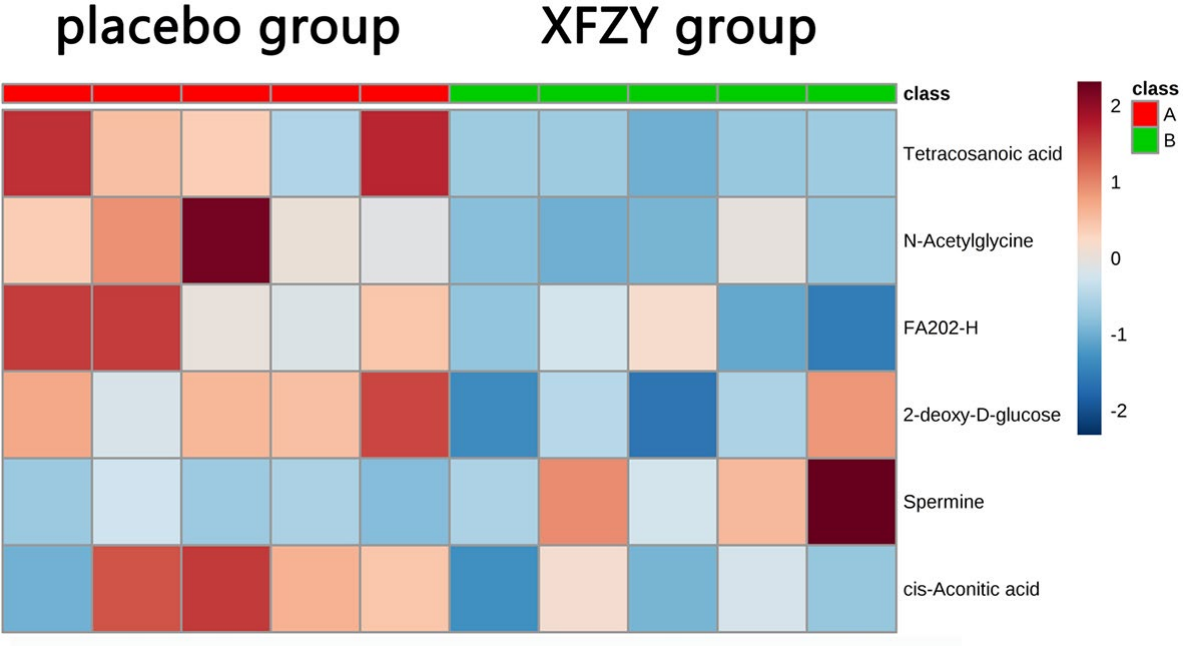
The six differential metabolites in the two groups are shown in the heat map using MetaboAnalyst 4.0 (A: placebo group; B: Xuefu Zhuyu decoction group). The row represents the metabolites, and the column represents the individual samples. Red bands indicate upregulated metabolites, and blue bands indicate downregulated metabolites in the two groups. The deeper the color, the greater the difference in metabolites.

As shown in **Table 3**, the differential metabolites related to FA metabolism were tetracosanoic acid (24:0) and FA (20:2)-H, which were obtained by election spray ionization negative (ESI–) mode with a mass of 367.358 and 307.264, respectively. Compared with the placebo group, tetracosanoic acid (24:0) and FA (20:2)-H in the XFZY decoction group were decreased, exhibiting that the ratio (XFZY/placebo) of tetracosanoic acid (24:0) or FA (20:2) was 0.46 (VIP = 2.40596, *P* = 0.008) or 0.78 (VIP = 2.21854, *P* = 0.020). 2-DG and cis-aconitic acid, which are related to glucose metabolism and obtained by ESI– mode, were downregulated with XFZY decoction treatment. The mass, VIP value, *P* value, and FC (XFZY/placebo) of 2-DG were 163.061, 2.03138, 0.039, and 0.50, respectively. The mass, VIP value, *P* value, and FC (XFZY/placebo) of cis-aconitic acid were 173.009, 1.9734, 0.048, and 0.81, respectively. The differential metabolites associated with amino acid metabolism were N-acetylglycine and spermine obtained by ESI+ mode. The concentration of N-acetylglycine in the XFZY decoction group was decreased as compared with the placebo group, which showed that the ratio (XFZY/placebo) of N-acetylglycine was 44% (VIP = 2.28626, *P* = 0.015). However, the concentration of spermine was increased by 208% compared with that in the placebo group (VIP = 1.99192, *P* = 0.045). As shown in **Figure 2**, the heat map illustrated total differential metabolites between the XFZY decoction group and placebo group: tetracosanoic acid, N-acetylglycine, FA, 2-DG, and cis-aconitic acid were downregulated, whereas spermine was upregulated.

**Table 3:** Differential metabolites between the XFZY group and placebo group patients from LC-MS/MS analysis.

Differential metabolite	Related metabolism	ESI mode	Mass (m/z)	RT (min)	FC (XFZY/placebo)	VIP	*P* value
Tetracosanoic acid (24:0)	Fatty acid metabolism	Neg	367.358	14.85	0.46	2.40596	0.008
N-acetylglycine	Amino acid metabolism	Pos	118.050	8.29	0.44	2.28626	0.015
FA(20:2)-H	Fatty acid metabolism	Neg	307.264	13.25	0.78	2.21854	0.020
2-deoxy-D-glucose	Glucose metabolism	Neg	163.061	1.04	0.50	2.03138	0.039
Spermine	Amino acid metabolism	Pos	203.223	8.76	3.08	1.99192	0.045
cis-aconitic acid	Glucose metabolism	Neg	173.009	0.87	0.81	1.9734	0.048

XFZY, Xuefu Zhuyu decoction; ESI, election spray ionization; RT, retention time; VIP, variable importance in the projection; P, probability; FC, fold change; Pos, positive; Neg, negative; FA, fatty acid.

**Figure 3:**
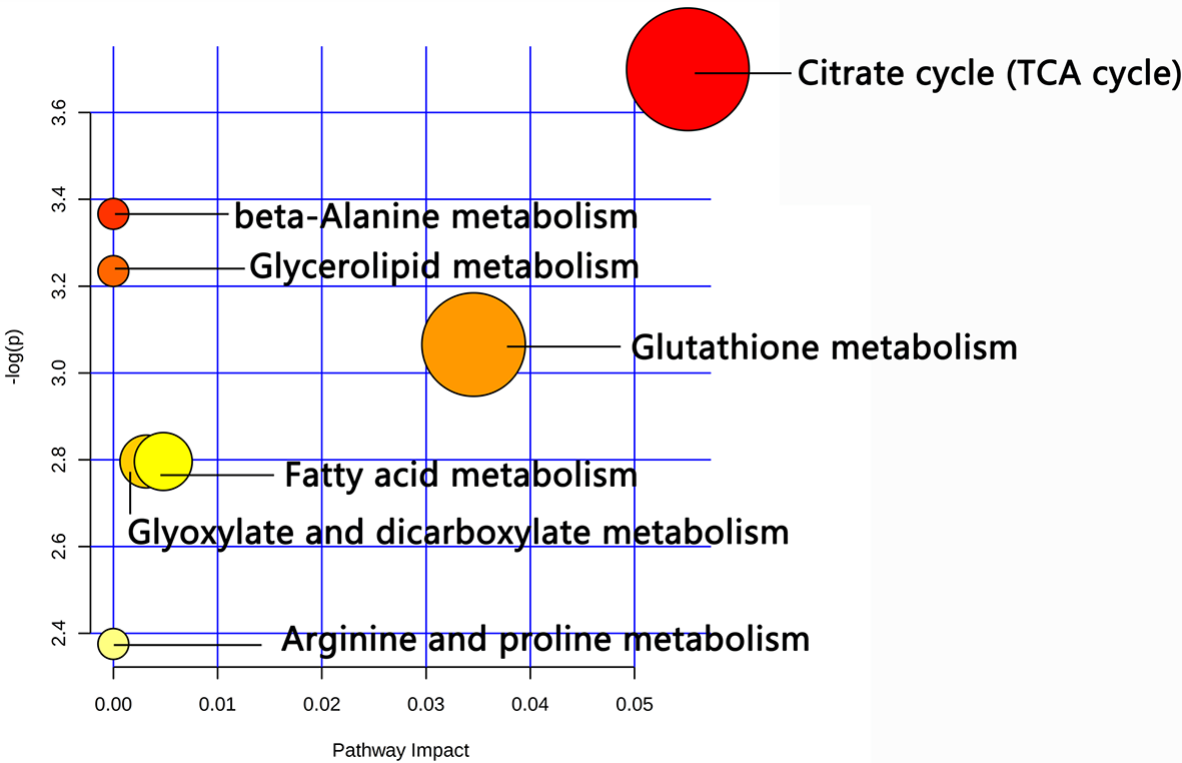
The disturbed metabolic pathways showed differential metabolites changed in the Xuefu Zhuyu decoction group as compared with the placebo group by MetaboAnalyst 4.0 software. Node radius was based on pathway impact values. Node color was based on *P* value.

MetaboAnalyst 4.0 software was used to analyze and predict the potential metabolic pathways involved in differential metabolites with XFZY decoction treatment. As shown in **Figure 3**, six differential metabolites were involved in seven metabolic pathways: citrate cycle (TCA cycle), β-alanine metabolism, glycerolipid metabolism, glutathione metabolism, glyoxylate and dicarboxylate metabolism, FA metabolism, and arginine and proline metabolism.

Further studies showed that cis-aconitic acid was involved in the TCA cycle as well as in glyoxylate and dicarboxylate metabolism. Spermine was predicted to participate in glutathione metabolism, arginine and proline metabolism, and β-alanine metabolism, respectively. FA (20:2) were predicted to be involved in FA metabolism and glycerolipid metabolism (**Table 4**).

**Table 4:** Differential metabolic pathway between the XFZY group and placebo group.

Pathway name	Match status	Match metabolites
Citrate cycle (TCA cycle)	1/20	cis-aconitic acid
β-alanine metabolism	1/28	Spermine
Glycerolipid metabolism	1/32	Fatty acid
Glutathione metabolism	1/38	Spermine
Glyoxylate and dicarboxylate metabolism	1/50	cis-aconitic acid
Fatty acid metabolism	1/50	Fatty acid
Arginine and proline metabolism	1/77	Spermine

XFZY, Xuefu Zhuyu decoction; TCA cycle, tricarboxylic acid cycle; match status, the number of accumulated metabolites/the number of all metabolites in metabolic pathways.

## Discussion

CHD is the leading cause of morbidity and mortality throughout the world (Lozano et al., 2012). The high mortality rate of CHD asks for effective complementary and alternative therapy to improve the prognosis of CHD patients. TCM based on syndrome differentiation is one of the effective complementary and alternative therapies. XFZY decoction, a classical traditional Chinese medication that regulates Qi and promotes blood circulation, is confirmed to be of clinical benefit for CHD treatment; however, the underlying mechanism is still unknown. The key pathogenesis of CHD is based on the abnormal metabolism of myocardial substrates and the disturbance of energy metabolism induced by myocardial ischemia or hypoxia (Doenst et al., 2013). A recent study found that metabolites involved in the TCA cycle and amino acid metabolism (tryptophan, arginine, and proline metabolism) were significantly decreased in fasting morning urine of CHD patients with Qi and Yin deficiency syndrome, suggesting that the metabolites in amino acid metabolism and TCA cycle were partially the material foundation of Qi (Zhou et al., 2019). In TCM theory, Qi is the commander of blood. Qi replenishment causes the promotion of blood circulation, whereas Qi stagnation leads to blood stasis, which suggests that Qi plays a leading role in CHD with Qi stagnation and blood stasis. In our study, the effect of XFZY decoction on low-molecular-weight metabolites was investigated by nontargeted metabolomics analysis. Compared with the placebo group, 513 kinds of metabolites were detected, and six metabolites were significantly differential in the serum from CHD patients after treatment with XFZY decoction. Among the six differential metabolites, FA (20:2)-H and tetracarboxylic acid (24:0), which are involved in FA metabolism; cis-aconitic acid, which participates in the TCA cycle; 2-DG, which is involved in glucose metabolism; and N-acetylglycine, which is involved in amino acid metabolism, were decreased, whereas spermine, which is involved in amino acid metabolism (such as arginine and proline, glutathione, and β-alanine metabolism), was increased as compared with the placebo group. This suggests that those low-molecular-weight metabolites might be the material foundation of XFZY decoction for regulating Qi and promoting blood circulation in CHD treatment.

### FA Metabolism

Because of continuous mechanical work, the heart has a high rate of ATP utilization. Because the high-energy phosphate pool in the heart is significantly small and could be exhausted within a few seconds, cardiomyocytes are very sensitive to changes in energy metabolism, and cardiac work strongly depends on ATP generation. Nearly 70% to 90% of ATP is produced by the oxidation of FA in cardiomyocytes (Doenst et al., 2013). Free FA are esterified to fatty acylCoA in the cytosol and are transported into the mitochondria matrix by carnitine. There, they form Acyl-CoA to enter β-oxidation (Berndt et al., 2019). Xu et al. found that FA and carnitine in the plasma of CHD patients were decreased compared with controls (Xu et al., 2016). However, others detected the lipid profile of H9c2 cells and then found that different types of FA were increased or decreased in hypoxic cardiomyocytes, which demonstrates that FA (18:1) was decreased, whereas FA (16:0) and FA (18:0) were increased as compared with controls. This indicates that different FA might change differently in special periods of hypoxia or ischemia in cardiomyocytes (Sousa et al., 2016). FA (20:2)-H and tetracarboxylic acid (24:0) are very-long-chain FA and should be oxidized by peroxisome to form medium- or short-chain FA. They are then transported to the mitochondria matrix by carnitine for β-oxidation. This means that FA (20:2)-H and tetradecanoic acid (24:0) provide substrates for β-oxidation, and the decrease in carnitine indicates the limitation of FA β-oxidation. In this study, we found that FA (20:2)-H and tetradecanoic acid (24:0) were downregulated, whereas carnitine did not decrease after XFZY decoction treatment as compared with the placebo group, suggesting that XFZY decoction might promote FA β-oxidation, which led to the decrease of FA in the serum.

### Glucose Metabolism

In myocardial metabolism, FA and glucose utilization is tightly linked and co-regulated. Utilization of one substrate may directly inhibit the utilization of the other, which is referred to as the “Randle cycle” (Randle, 1998). In ischemic myocardium, FA oxidation is limited, and the utilization of glucose is increased. As a glucose analogue, 2-DG inhibited the critical enzymes of glycolysis (hexokinase and phosphoglucose isomerase), which inhibited glycolysis with the downregulation of glucose-6-phosphate and fructose-6-phosphate. In addition, 2-DG possessed a mannose-like property, which competed with mannose during the initial steps of N-linked glycosylation, resulting in protein misfolding and endoplasmic reticulum stress (Xi et al., 2014). In rat models, 2-DG was encountered as cardiac toxicity, with microscopic findings of vacuolar degeneration and hypertrophy of the endothelial cells of the endocardium (Terse et al., 2016). Studies in tumors have found that at low doses, 2-DG mainly interfered with N-linked glycosylation, resulting in endoplasmic reticulum stress, apoptosis, and autophagy. At medium doses, 2-DG also blocked glycolysis, leading to ATP reduction, and the subsequent growth inhibition. At high doses, 2-DG started to disrupt the pentose phosphate pathway, which caused growth arrest and oxidative stress (Xi et al., 2014). It was found for the first time that 2-DG in the serum of CHD patients was decreased after treatment with XFZY decoction, which suggested that XFZY decoction could attenuate the inhibition of N-glycosylation and glycolysis; alleviate endoplasmic reticulum stress, apoptosis, and autophagy; promote ATP production; and protect cardiomyocytes by downregulating 2-DG.

Acetyl-CoA, a common end product of glucose and FA oxidation, enters the TCA cycle and provides energy for cardiomyocytes (Opie, 2004). Cis-aconitic acid is an important intermediate that participates in the TCA cycle. It combines with aconitase, which converts citric acid to isocitric acid, and maintains the progress of the TCA cycle. The decrease in cis-aconitic acid suggests the limitation of TCA cycle metabolism. Our study first found that cis-aconitic acid, one intermediate of the TCA cycle, had the same change as 2-DG. Compared with the placebo group, cis-aconitic acid was decreased after XFZY decoction treatment. Combined with the previous changes in FA metabolism, it suggested that the upregulation of FA metabolism might inhibit glucose metabolism, as shown in the decrease of the intermediate in the TCA cycle.

### Amino Acid Metabolism

Amino acids can also be used as energy metabolites. They form precursors of glucose and FA metabolism through deamination or transamination and participate in metabolic pathways, such as the TCA cycle. In addition, amino acid-derived bioactive substances play an important role in the cardiovascular system. Spermine is a kind of polyamine that is further produced by putrescine converted from arginine and has many physiological functions. It was reported that spermine was significantly decreased after myocardial ischemia/reperfusion injury in rats, suggesting that spermine may play a key role in myocardial ischemia/reperfusion injury (Han et al., 2009). Animal experiments showed that spermine enhanced the antioxidant status: spermine supplementation and extended spermine administration could promote the expression of antioxidant enzymes, such as glutathione reductase by upregulating nuclear factor erythroid 2-related factor 2 (Nrf2) expression (Cao et al., 2018). Arginine plays a critical antiatherogenic role in the development of cardiovascular disease by regulating endothelial cell homeostasis. Nitric oxide (NO), produced by the oxidation of arginine in endothelial cells, promotes beneficial effects in the vasculature, including vasodilation, enhanced fibrinolysis, and inhibition of multiple atherothrombotic biological processes, such as platelet aggregation, leukocyte adhesion, endothelin generation, and smooth muscle cell proliferation (Nausch et al., 2008; Tang et al., 2009). β-alanine can be transaminated to pyruvate, providing a substrate for cardiac glucose oxidation. In our study, we found for the first time that the serum spermine concentration in CHD patients was increased after treatment with XFZY decoction. Bioinformatics analysis further indicated that spermine was involved in glutathione metabolism, arginine and proline metabolism, and β-alanine metabolism. It was suggested that XFZY decoction could alleviate oxidative stress in cardiomyocytes by promoting glutathione metabolism, maintain endothelial function by upregulating arginine metabolism, and increase the production of aerobic oxidative substrates in cardiomyocytes by promoting β-alanine metabolism.

In our study, we further investigated the mechanism of XFZY decoction for regulating Qi and promoting blood circulation from the perspective of biological functions of low-molecular-weight metabolites. Some studies have shown that XFZY decoction alleviated cardiomyocyte apoptosis and oxidative stress (Meng et al., 2018) as well as the inhibition of platelet aggregation (Li et al., 1999). For the first time, we found that 2-DG was decreased whereas spermine was increased in the serum of CHD patients after treatment with XFZY decoction. Through exploration of the effects of those two differential metabolites on ischemic myocardium, we confirmed the potential mechanism of XFZY decoction in CHD treatment: the decrease of 2-DG alleviated apoptosis and autophagy and promoted glycolysis to generate ATP after treatment with XFZY decoction, and the increase in spermine alleviated apoptosis and oxidative stress and inhibited platelet aggregation in the XFZY decoction group, which explained the similar functions of XFZY decoction in previous animal experiments. The elevation of spermine also illustrated the increase of arginine metabolism, which might generate more NO to alleviate coronary atherosclerosis. In addition, the decrease in 2-DG promoted ATP synthesis. Because NO and ATP are regarded as a part of the involvement of Qi in TCM theory (Deng et al., 2012; Chen et al., 2018), it is suggested that the metabolites of Qi might be associated with 2-DG and spermine.

### Limitation

Although we believe that our study investigated the effects of XFZY decoction on metabolic profiles in CHD patients and the results could partly explain the mechanism of XFZY decoction for regulating Qi and promoting blood circulation, some limitations should still be considered. One limitation is that the sample size in this study is small. Another limitation is that the follow-up time is not long enough, so we did not focus on the study of the primary or second end points. Because there was statistical difference between the placebo group and the XFZY decoction group before treatment, we excluded the metabolites that have similar trends in our analysis. In addition, metabolomics itself has some limitations. Because of the wide concentration range and chemical diversity of metabolites, there is no instrument to detect all metabolites in a single analysis. In this study on metabolomics of CHD, we detected the metabolites in the serum. However, the metabolites in the circulatory system may originate from nonmyocardial tissues. The use of metabolic drugs and the excretion function of the liver and kidney may also affect the level of metabolites. Those two reasons increased the uncertainty of applying metabolomics to clinical detection of CHD (Dias and Koal, 2016). Moreover, some energy metabolites, such as ATP, adenosine diphosphate (ADP), and phosphocreatine were not detected in this study. Considering the limitation of nontargeted metabolomics, we should optimize the methods, such as choosing targeted metabolomics in the future.

## Conclusion

In conclusion, we found that XFZY decoction caused the changes in six metabolites in FA, glucose, and amino acid metabolism with the detection of the serum in CHD patients by nontargeted LC-MS/MS analysis. We further predicted seven metabolic pathways according to those differential metabolites. Among them, 2-DG and spermine might partially be the material foundation of Qi in TCM theory, which suggests potential targets of XFZY decoction for regulating Qi and promoting blood circulation in CHD treatment.

## Data Availability

The datasets generated for this study are available on request to the corresponding author.

## Ethics Statement

The studies involving human participants were reviewed and approved by the Ethics Review Committee of Chinese PLA General Hospital (No. S2015-048-01). The patients/participants provided their written informed consent to participate in this study.

## Author Contributions

XL conceived, designed, and supervised the clinical trial and experiments and contributed to manuscript revision. TT performed the trials and statistical analysis and drafted the manuscript. TH coperformed the trials. XW coperformed the statistical analysis. All authors read and approved the submitted version.

## Funding

This work was supported by the National Basic Research Program of China (XL: No. 2015CB554402 and 2015CB554405) and the National Natural Science Foundation of China (XL: No. 31771287).

## Conflict of Interest Statement

The authors declare that the research was conducted in the absence of any commercial or financial relationships that could be construed as a potential conflict of interest.
