# Supplementary material for: Metabolic Profiling Analysis of Patients With Coronary Heart Disease Undergoing Xuefu Zhuyu Decoction Treatment
Source: Front Pharmacol. 2019 Sep 10;10:985. doi: 10.3389/fphar.2019.00985 (PMC6746894; doi:10.3389/fphar.2019.00985)
Supplement: Supplementary file 1 [file DataSheet_1.doc]

Supplementary Material

## Supplementary Figures


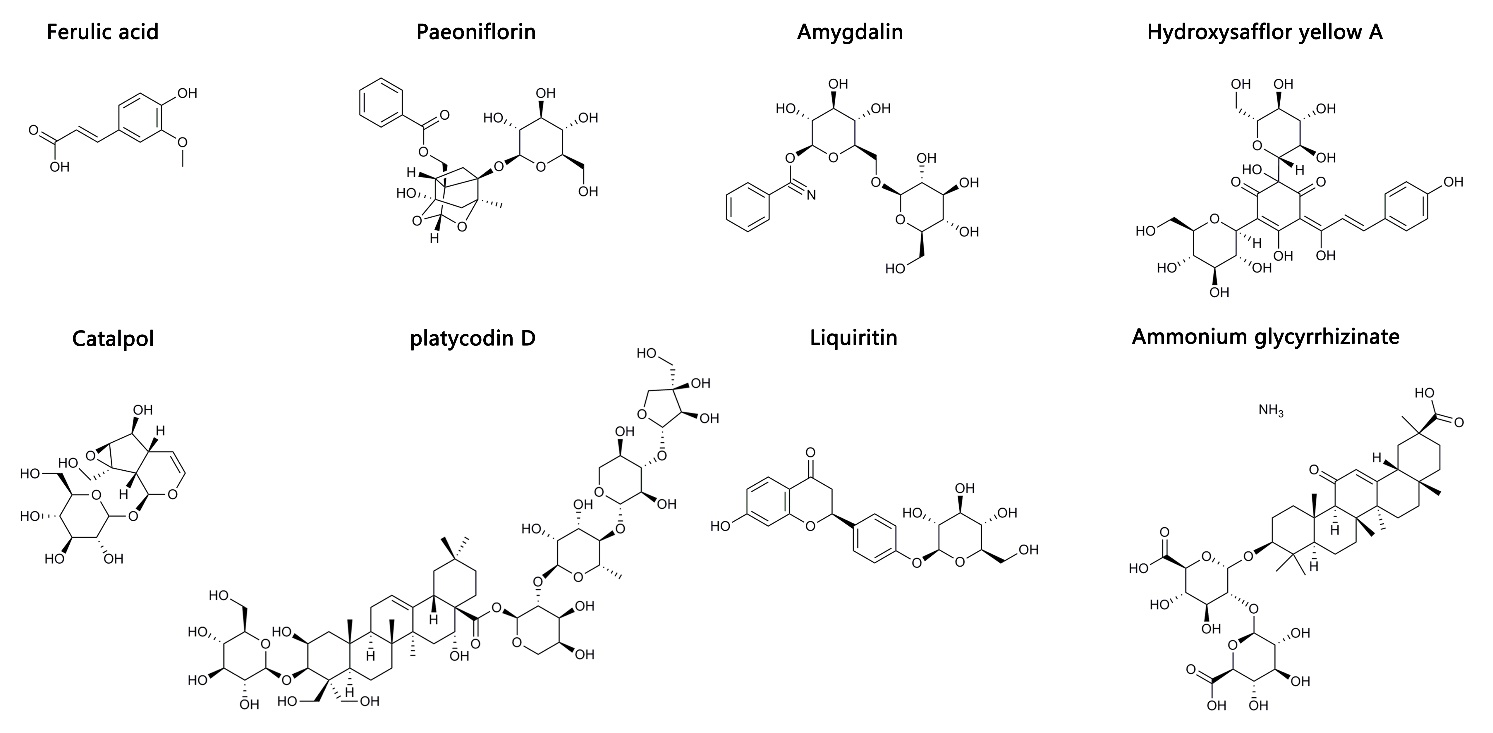


**Supplementary Figure 1.** The chemical structures of the main chemical components of Xuefu Zhuyu Decoction.


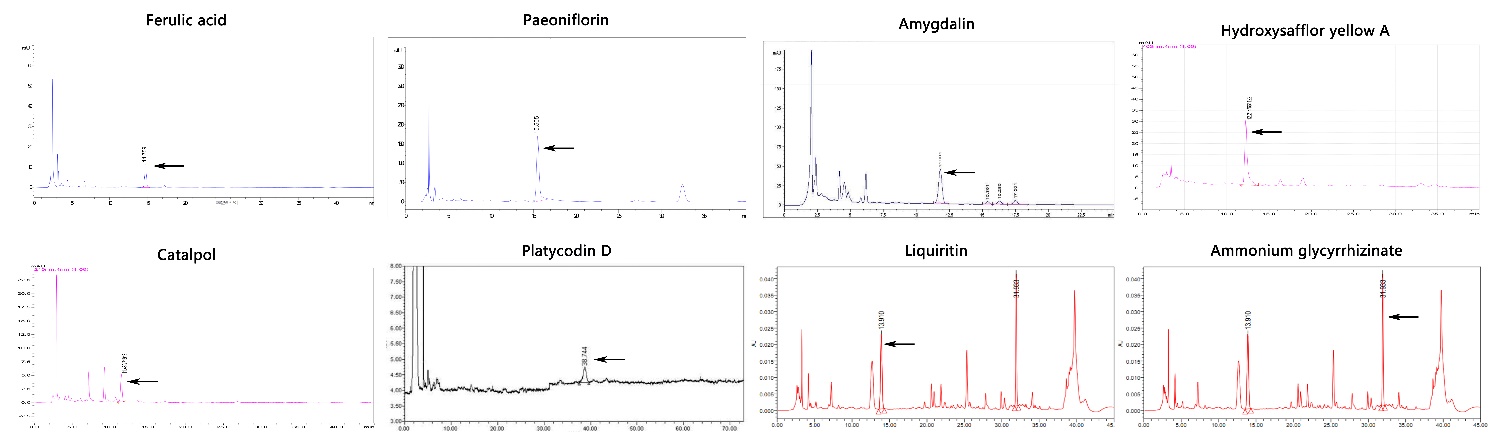


**Supplementary Figure 2.** The high-performance liquid chromatography (HPLC) profile of the main chemical components of Xuefu Zhuyu Decoction performed by the manufacturer.
